# Effect of the pectin contents and nanostructure on the stem straightness of two *Paeonia lactiflora* cultivars

**DOI:** 10.7717/peerj.15166

**Published:** 2023-04-13

**Authors:** Yiran Huang, Anqi Ren, Yingling Wan, Yan Liu

**Affiliations:** 1 Beijing Key Laboratory of Ornamental Plants Germplasm Innovation & Molecular Breeding, Beijing, China; 2 National Engineering Research Center for Floriculture, Beijing, China; 3 Beijing Laboratory of Urban and Rural Ecological Environment, Beijing, China; 4 School of Landscape Architecture, Beijing Forestry University, Beijing, China

**Keywords:** *Paeonia lactiflora*, Pectin fraction, Nanostructure, Atomic force microscopy

## Abstract

Herbaceous peony (*Paeonia lactiflora* Pall.) is an ancient ornamental crop and, in recent decades, an emerging popular cut flower. Straight stems are a vital criterion for cut herbaceous peony selection, while many cultivars bend as the plant develops. Pectin helps maintain the mechanical strength of the cell wall. However, little is known about its role in the stem bending of herbaceous peony. Two herbaceous peony cultivars with contrasting stem morphologies (‘Dong Fang Shao Nv’, upright; ‘Lan Tian Piao Xiang’, bending gradually) at five developmental stages were used as materials to investigate the effects of pectin content and nanostructure on straightness using the carbazole colorimetric method and atomic force microscopy observations. The contents of water-soluble pectin (WSP), CDTA-soluble pectin (CSP), and sodium carbonate-soluble pectin (SSP) differed significantly between the two cultivars, and the contents and angle of the flower and branch showed correlations. For the pectin nanostructure, WSP showed agglomerates and long chains, with a higher proportion of broad agglomerates at the later stages of the bending cultivar than the upright cultivar. CSP showed branched chains, and the proportion of broad chains was higher in the upright cultivar at later stages, while CSP shape changed from agglomerates to chains in the bending cultivar. SSP mainly consisted of short linear main chains, and side chains in the upright stem were stacked, and the bent cultivar had more broad and short chains. It can be concluded that the contents, nanometric shape, and size of the three kinds of pectin are highly likely to affect herbaceous peony stem straightness. This study provides a theoretical basis for the role of pectin in the production and breeding of herbaceous peony cut flowers.

## Introduction

Herbaceous peony (*Paeonia lactiflora* Pall.) is extensively cultivated around the world for its beautiful brightly colored flowers (Wang et al., 2022). Moreover, herbaceous peony was planted in ancient China in courtyards to appreciate its curved stems. In recent decades, it has become one of the most popular new cut flowers, and high transaction prices have brought herbaceous peony farmers of significant economic benefits (Kamenetsky-Goldstein & Yu, 2022). A straight stem is a vital criterion for cut peony selection (Wan et al., 2020). In field observations, it was determined that stems of a large number of cultivars would gradually bend along with the development, which became a nonnegligible limit to high-quality cut herbaceous peonies. Therefore, it is urgent to investigate the underlying mechanism of stem bending to accelerate industrial development.

Cellulose, hemicellulose, lignin, and pectin are the main components that provide the mechanical strength of the cell wall (Chen et al., 2021; Shi et al., 2022b; Somerville et al., 2004), and they are the main components of herbaceous peony stem straightness studies. Among them, lignin has been studied the most. The total lignin content was correlated with stem straightness, and the S-lignin and G-lignin monomer contents and the ratio of S/G lignin and lignin distribution area of the upright cultivars were higher than those of cultivars with poor straightness (Wan et al., 2020; Tang et al., 2022; Zhao et al., 2020a; Zhao et al., 2020b). Exogenous sprays of silicon, calcium, the calcium ion chelator EGTA, and melatonin affected the mechanical strength of herbaceous peony stems by influencing the secondary cell wall thickness, lignin content, and gene expression of lignin monomer synthesis pathways and deposition, such as *PAL*, *C4H*, *4CL*, *CCR*, *CAD*, *CSE*, *POD*, *COMT*, and *CCoAOMT* (Zhao et al., 2013; Tang et al., 2019; Zhao et al., 2019; Zhao et al., 2021; Zhao et al., 2022). Similar findings have been found in studies of maize, oilseed rape, gerbera cut flower and other plants (Alikhani et al., 2021; Hu et al., 2022; Liu et al., 2022). However, it has also been argued that the cellulose content is associated with straightness. Some researchers thought that cellulose content was not strictly correlated with herbaceous peony straightness (Zhao et al., 2012). Our group found that the cellulose content was highly significantly and positively correlated with the stem tilt angle (the angle between the branch and the horizontal plane) in herbaceous peony cultivars with contrasting straightness (*R* = 0.955 and 0.873). The more upright the cultivar is, the higher the cellulose content (Zhao et al., 2015). We also found significant differences in hemicellulose content between upright and bending stems of *P. lactiflora* at S3 and S5 (Wan et al., 2020). In addition, pectin content was correlated with herbaceous peony straightness, where water-soluble pectin (WSP) content was significantly and negatively correlated with straightness at later stages of development (Hou et al., 2022). There are conflicting claims on the relevance of protopectin to straightness. Li (2013) concluded that protopectin was highly significantly and positively correlated with stem strength, while Hou et al. (2022) noted that herbaceous peony straightness was negatively correlated with protopectin content at the middle stage of development. Pectin content was also found to correlate with stem bending in other plants, such as ramie, gerbera, and sugarcane (Zhou, 2017; Li, 2019; Cheng et al., 2020). Previous studies have indicated that plants can be collapsed by regulating pectin modification-related genes (Xiao et al., 2017; Miller et al., 2016). In contrast, there is no systematic and in-depth study on pectin in herbaceous peony.

Pectin is mainly present in the middle lamella and primary cell walls and makes adjacent cells stick together. Moreover, modification of pectin affects gelation (Daher & Braybrook, 2015; Satya et al., 2020; Zhang et al., 2020). Pectin is divided into WSP, CDTA-soluble pectin (CSP), and sodium carbonate-soluble pectin (SSP) according to the type of solvent (Zhang et al., 2020). In addition, previous studies have shown that polysaccharide degradation, especially changes in pectin solubility and rheological properties, leads to cell wall softening, thereby causing softening of the fruits (Brummell, 2006; Shi et al., 2022a; Uluisik & Seymour, 2020). Studies on mangoes, strawberries, blood oranges, and figs showed that the softening process was followed by an increase in WSP content and a decrease in CSP and SSP contents (Cardenas-Perez et al., 2018; Frempong et al., 2022; Ren et al., 2022; Zhang et al., 2020). Further studies showed that changes in pectin aggregate size, number, width, and length of chains at the nanometric scale were also closely related to the fruit ripening and softening process (Deng et al., 2019; Paniagua et al., 2017a; Pieczywek et al., 2020). To the best of our knowledge, little is known about the role of pectin nanostructures in stem straightness.

Our objective was to investigate whether pectin content and nanostructure differed among herbaceous peony cultivars with different straightness and whether they were correlated with straightness. Two cultivars with contrasting straightness at five stages were used, and the contents of WSP, CSP, and SSP and nanostructural changes in pectin were investigated. The results of this manuscript would serve as a basis to understand the mechanism of stem straightness at the cytological level and provide a theoretical basis for the cultivation and breeding of high-quality cut herbaceous peonies.

## Material and Methods

### Plant materials and growth conditions

**Figure 1:**
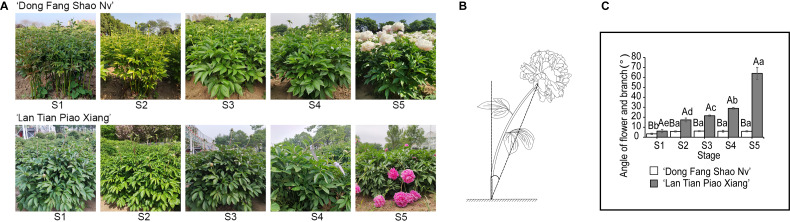
The stem characteristics of ‘Dong Fang Shao Nv’ and ‘Lan Tian Piao Xiang’. (A) Plant growth of two cultivars at five stages. (B) Schematic diagram of the angle of flower and branch measurement. (C) Changes in the angle of flower and branch of two herbaceous peony cultivars at five stages. Data in the graph are means ± standard deviations. Uppercase letters in C indicate comparisons between different cultivars at the same stage, and lowercase letters indicate comparisons between different stages of the same cultivar. Different lowercase letters among groups mean significant differences at *P* < 0.05.

Two cultivars of *P. lactiflora*, ‘Dong Fang Shao Nv’ and ‘Lan Tian Piao Xian’, were used in this study. The cultivars were both eight-year-old plants obtained from the Caozhou Peony Garden, Heze, China (35.14° N, 115.26° E) with normal and identical fertilization and water. The parent materials were spaced approximately 82 cm apart and 106 cm apart in rows. The plants were fertilized once a year in October or November and once every two months with a compound fertilizer for foliar application, combined with insect removal. Daily maintenance was mainly for diligent weeding and loosening the soil for air. Six plants were randomly selected for measurement at each stage. From stem elongation to flowering, every seven days was one period and five stages were divided as described by Wan et al. (2020). The growth conditions of ‘Dong Fang Shao Nv’ and ‘Lan Tian Piao Xiang’ at five stages are shown in Fig. 1A, stage S1 to S5 corresponding to the stem elongation, bud conception, color transmission, bud softness, and flowering stages of herbaceous peony, respectively. Samples from S1 to S5 were obtained by cutting 5–10 cm stem segments under the flower. All the samples were quickly frozen in liquid nitrogen and stored in a −80 °C freezer for subsequent use. At each stage, to measure the degree of stem bending, the angle of the flower and branch was measured. First, the flower was connected with the base of the stem, and second, a plumb line was extended from the base of the stem. These two lines formed a pinch angle, which was called the angle of the flower and branch. This definition was based on a previous study (Wan et al., 2020), and the schematic diagram is shown in Fig. 1B.

### Determination of pectin fractions in the cell wall

The cell wall material (CWM) was extracted first as described in a previous study (Wan et al., 2020). Three hundred milligrams of stem segments were weighed and added to 1 mL of 80% ethanol solution and homogenized rapidly at room temperature. Then, the samples were placed in a water bath at 90 °C for 20 min. After cooling to room temperature, the mixtures were centrifuged at 6000 × g for 10 min at 25 °C, and the supernatant was discarded. The precipitate was washed with 1.5 mL of 80% ethanol and propanol successively, vortexed for approximately 2 min followed by centrifugation at 6000 × g 25 °C for 10 min, and the supernatant was discarded. Subsequently, 1 mL of 90% dimethyl sulfoxide solution was added to remove the starch for 15 h, and the centrifugation step was repeated. The final precipitate was dried and weighed as CWM. The CWM was weighed to 3 mg, and the carbazole colorimetric method was used to measure the pectin content of the three solubles. The reagent kit was purchased from Suzhou Kemin Biotechnology Corporation, and the manufacturer’s instructions were followed. All measurements were performed with three biological replicates.

The different kinds of pectin fractions were extracted from stems according to the method described by Fishman et al. (1993) and Wang et al. (2021) with appropriate modifications. The CWM in the pectin fraction determination step was alcohol insoluble residue (AIR) (Renard Catherine, 2005). A 0.05 g AIR sample was mixed with distilled water, shaken for 1 h at 25 °C, repeated three times, and then centrifuged at 4 °C and 10,000 × g for 15 min. The supernatant was obtained as the WSP extract and diluted 6 times for the measurement. The residue was transferred to 15 mL of cyclohexane-trans-1,2-diamine tetraacetic acid (CDTA, 0.05 M, pH 6.5 adjusted by sodium acetate) solution. The sample was oscillated for 6 h at 25 °C, centrifuged for 15 min (10,000 g, 4 °C), filtered and diluted 6 times to use as CSP. For SSP fractions, the remaining residue was incubated with 15 mL of 0.05 M Na_2_CO_3_ (containing 0.002 M CDTA) for 16 h at 4 °C and stirred for 6 h at 25 °C. After centrifugation and filtration, the resulting supernatant was diluted 6 times and left to be measured.

### Observation of the pectin nanostructure by atomic force microscopy (AFM)

A total of 10 mL of diluted sample solutions was dropped onto freshly cleaved mica and then left at room temperature for approximately 15 min until the sample dried. The nanostructure of the pectin fractions was scanned by an atomic force microscope (BRUKER MULTIMODE 8, USA), and the probe was for Bruker’s intelligent scanning, model SCANASYST-AIR. The scanning resolution was 512 × 512 points. At least three images were observed at the scale of 10 by 10 µm^2^ under AFM. To show the nanoscale structure of pectin more clearly, the image size shown in this manuscript is 2.5 × 2.5 µm^2^ or 5 × 5 µm^2^.

### AFM image analysis

The images were processed using offline software (NanoScope Analysis version 1.40) for qualitative and quantitative information about pectin nanostructures as described by Paniagua et al. (2017a) and Pan et al. (2018). The “Flatten” function in the software corrected for height deviations due to drift in the Z-voltage of the scanning tube or the tilt of the sample itself, as well as the bow of the scanning tube. Since the observed pectin contained multiple morphologies, they were characterized by measuring the agglomerate width and the chain structure length. Using the ‘Section Analysis’ function in the NanoScope Analysis offline software, we measured the width and length of the chain by drawing a cross-cutting line vertically on the image or along the direction of the chain. The length of a single chain was defined as the total length of the trunk and branches (Adams et al., 2003). The data were plotted as violin plots based on the specific values of the measured molecular lengths and widths. To ensure reliable experimental results, at least 90 chains were measured at each developmental stage.

### Statistical analysis

The effects of cultivars and stages and their interaction on pectin content with two-way ANOVA and the statistical significance of the differences with Tukey’s test were analyzed in SPSS 25.0.0.0. Differences in indicators were tested by t-tests, and correlations were analyzed by Spearman correlation. Violin plots were generated in R 4.2.1 (R Core Team, 2022).

## Results

### Changes in angle between flower and branch

One of the most intuitive indicators to reflect stem straightness is the angle of the flower and branch. As shown in Fig. 1C, the angle of the flower and branch of ‘Dong Fang Shao Nv’ varied from 3.38° to 6.12° at five stages, and the stem remained upright. There was no significant difference in the angle of the flower and branch from S2 to S5 except for S1, when the angle of the flower and branch was significantly smaller. The angle of the flower and branch of ‘Lan Tian Piao Xiang’ gradually increased, ranging from 6.13° to 64.05° and were significantly different during the five stages. Bent stems were observed from S4 to S5. At all the stages, the angles of ‘Lan Tian Piao Xiang’ were significantly greater than those of ‘Dong Fang Shao Nv’, and the difference reached a maximum at S5. Thus, ‘Dong Fang Shao Nv’ and ‘Lan Tian Piao Xiang’ can be used as representatives of upright and bending herbaceous peonies, respectively.

### Quantitative analysis of cell wall pectin contents

**Figure 2:**
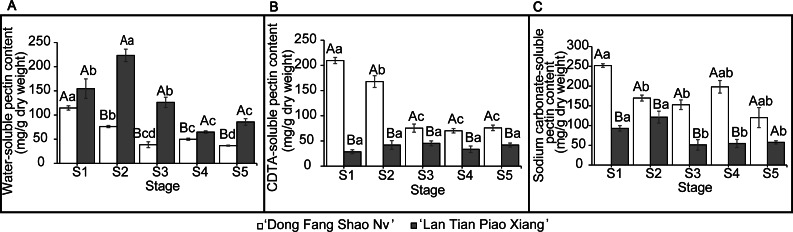
Contents of WSP (A) CSP (B) and SSP (C) in ‘Dong Fang Shao Nv’ and ‘Lan Tian Piao Xiang’ at S1–S5. Data in the graph are means ± standard deviations. Uppercase letters in each panel indicate comparisons between different cultivars at the same stage, and lowercase letters indicate comparisons between different stages of the same cultivar. Different letters among groups mean significant differences at *P* < 0.05.

The contents of WSP, CSP, and SSP in the two cultivars are shown in Fig. 2. The WSP content of ‘Lan Tian Piao Xiang’ was significantly greater than that of ‘Dong Fang Shao Nv’ from S2 to S5, and the content of ‘Lan Tian Piao Xiang’ was 2.93 times greater than that of ‘Dong Fang Shao Nv’ at S2 when the difference between the two cultivars was the largest. The WSP of the two cultivars showed different trends with development, where the content of ‘Dong Fang Shao Nv’ at S1 was significantly greater than that at the other stages, while the content of ‘Lan Tian Piao Xiang’ increased first at the early stage and peaked at S2. ‘Dong Fang Shao Nv’ had a consistently higher CSP content than ‘Lan Tian Piao Xiang’ during stem development, and the largest difference (i.e., 7.36 times) was shown at S1. ‘Dong Fang Shao Nv’ had the highest CSP content at S1 and subsequently decreased, while the CSP content of ‘Lan Tian Piao Xiang’ was always at a low level. Moreover, the SSP content of ‘Dong Fang Shao Nv’ was significantly higher than that of ‘Lan Tian Piao Xiang’ from S1 to S4, and the largest difference occurred at S1, with a difference of 2.71 times. The SSP content of ‘Dong Fang Shao Nv’ changed with development, and the SSP content of ‘Lan Tian Piao Xiang’ remained high only at S1 and S2. The two-way ANOVA test, shown in Table 1, demonstrated that both the cultivar and development stage had significant effects on the pectin content of the three solubilities.

### Correlation analysis of pectin content and angle between flower and branch

Correlation analysis between the three pectin contents of the two cultivars and the angles of flower and branches at different periods was conducted to analyze how the cell wall pectin contents affected the straightness of herbaceous peony stems and the results are shown in Table 2. The content of WSP was highly significant and positively correlated with the angle of flower and branch at S1 (*P* < 0.01) and positively correlated at S2 (*P* < 0.05), indicating that cultivars with relatively higher WSP contents had a greater angle between flower and branch and were more likely to bend. Contents of CSP were highly significant and negatively correlated with the angle of the flower and branch at S1 and S2 (*P* < 0.01). It was also demonstrated that the CSP content was greater in the upright cultivar at S1 and S2. The SSP content was negatively correlated with the angle of the flower and branch at all stages but was not significant (*P* > 0.05).

**Table 1:** Results of the influences of WSP, CSP and SSP contents on both cultivar and development three stage.

		*P*-value
WSP content	Cultivar	0.000
	Stage	0.000
	Cultivar × Stage	0.000
CSP content	Cultivar	0.000
	Stage	0.000
	Cultivar × Stage	0.000
SSP content	Cultivar	0.000
	Stage	0.000
	Cultivar × Stage	0.000

**Notes.**

In the table, *P* value, which was less than 0.05, indicates the statistical significance.

**Table 2:** Correlation analysis of pectin content and key structural indexes with branch angle of *Paeonia lactiflora*.

Factors	Correlation coefficient				
	S1	S2	S3	S4	S5
WSP content	0.943^**^	0.829^*^	0.657	0.771	0.657
CSP content	−0.886^**^	−0.943^**^	−0.771	−0.657	−0.714
SSP content	−0.714	−0.771	−0.771	−0.600	−0.771

**Notes.**

* The correlation coefficient is significant at the 0.05 level.

** The correlation coefficient is significant at the 0.01 level.

### AFM analysis of pectin fractions

**Figure 3:**
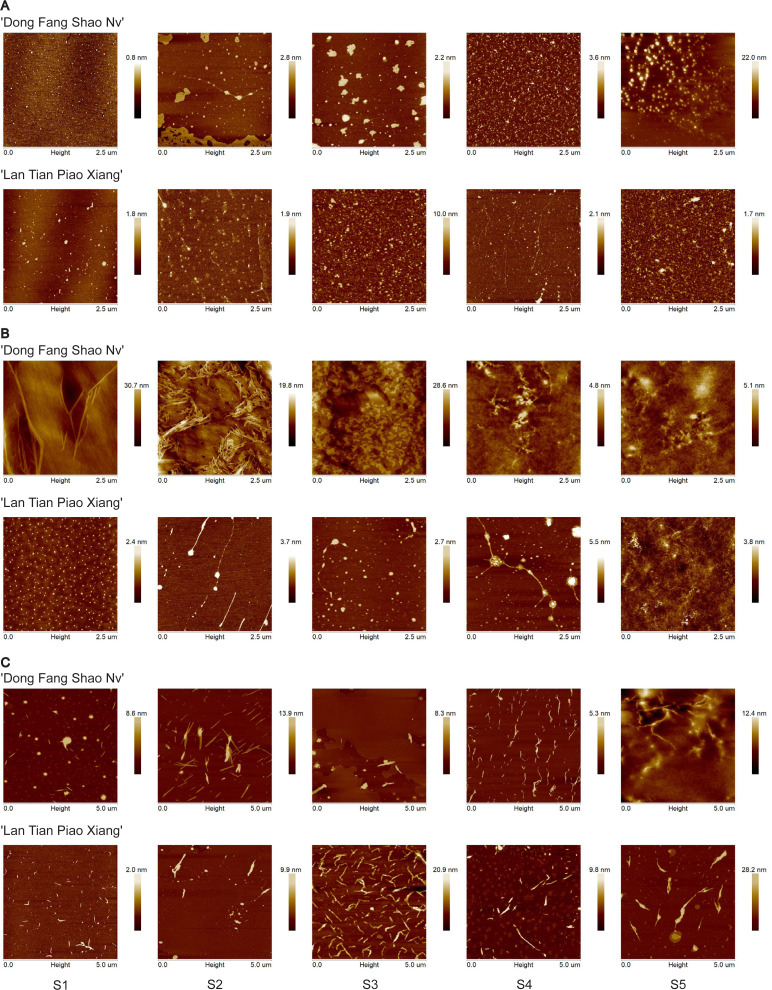
Representative topographical AFM images of WSP (A) CSP (B) and SSP (C) from cell walls of ‘Dong Fang Shao Nv’ and ‘Lan Tian Piao Xiang’ at S1–S5.

Representative topographical AFM images of WSP from ‘Dong Fang Shao Nv’ and ‘Lan Tian Piao Xiang’ at the five stages are shown in Fig. 3A. ‘Dong Fang Shao Nv’ showed an elliptic polymer shape, which tended to aggregate into chains at S2. Thin lamellar structures appeared at S2–S3 and were distributed around the agglomerates. ‘Lan Tian Piao Xiang’ showed multiple shapes at the five stages. The linear chains appeared at S2 and S4, and the chain became longer at S4, whereas the other stages retained oval aggregates.

CSP nanostructures of the two cultivars are presented in Fig. 3B. Long and branched chains were observed at S1 in ‘Dong Fang Shao Nv’, and intertwined and tightly aggregated chain structures occurred at S2. At S3, densely arranged linear chains appeared near the lamellar structure, which disappeared and was replaced with clear branched and intertwined chains at S4 and S5. In ‘Lan Tian Piao Xiang’, an equal size dot-like agglomeration structure with homogeneous distribution was shown. Then, chains were observed at S2 and S3. The branched structure occurred at S4 and changed at S5 into an entangled complex branched-chain structure, which was similar to that of ‘Dong Fang Shao Nv’ at S4.

The nanostructure of SSP in the two cultivars is shown in Fig. 3C. Short linear main chains with some agglomerates dispersed around were observed in both cultivars. ‘Dong Fang Shao Nv’ showed branched structures at S2, S4, and S5, and the chains were stacked at S4. In ‘Lan Tian Piao Xiang’, only a side chain structure from S1 to S3 was observed. The remaining stages were dominated by short chains that were widely dispersed.

**Figure 4:**
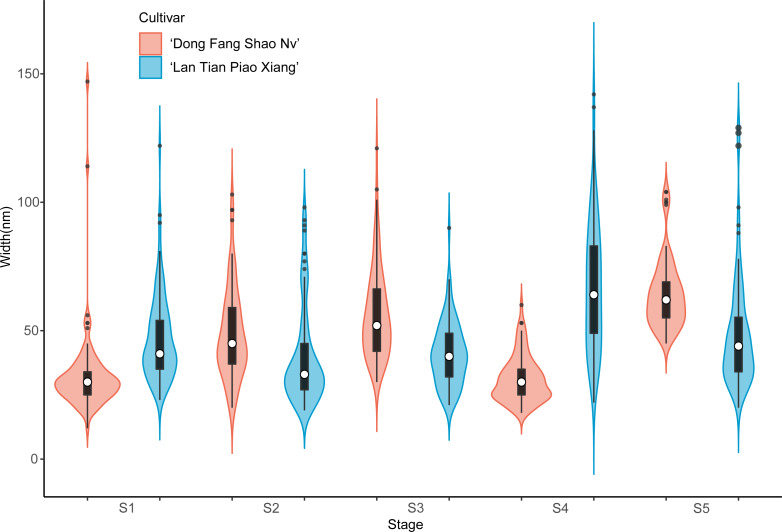
Violin plots showing the width distributions of WSP samples isolated from herbaceous peony stem cell walls at S1–S5. The colored areas indicate the frequency distribution of the width. The vertical black bars indicate the range of the mean value, where the white solid dots represent the median of the width. Black solid bar above and below the white solid circle indicates the upper and lower quartile, respectively. Dark scatters indicate outliers.

Further analysis of the width of WSP of the two cultivars was conducted by means of violin plots, and the results are shown in Fig. 4. At S1, most of the WSP widths were concentrated in the range from 12 to 56 nm in ‘Dong Fang Shao Nv’ (98%), while those of ‘Lan Tian Piao Xiang’ were concentrated in the range of 30–70 nm (83%). Therefore, the median WSP nanowidth of ‘Lan Tian Piao Xiang’ was significantly larger than that of ‘Dong Fang Shao Nv’. However, the result was different at S2–S3, when the median WSP width of ‘Dong Fang Shao Nv’ was larger than that of ‘Lan Tian Piao Xiang’. ‘Lan Tian Piao Xiang’ had more WSP agglomerates at S2, resulting in a higher number of outliers on the violin plot. The violin plot of ‘Lan Tian Piao Xiang’ at S4 in Fig. 4 was narrower and longer than that of ‘Dong Fang Shao Nv’, which suggested that ‘Lan Tian Piao Xiang’ had a broader WSP than ‘Dong Fang Shao Nv’ and had a more varied WSP width range (from 22 to 142 nm). In addition, ‘Lan Tian Piao Xiang’ showed more large outliers at S5; however, most of the WSP widths were still smaller than those of ‘Dong Fang Shao Nv’.

**Figure 5:**
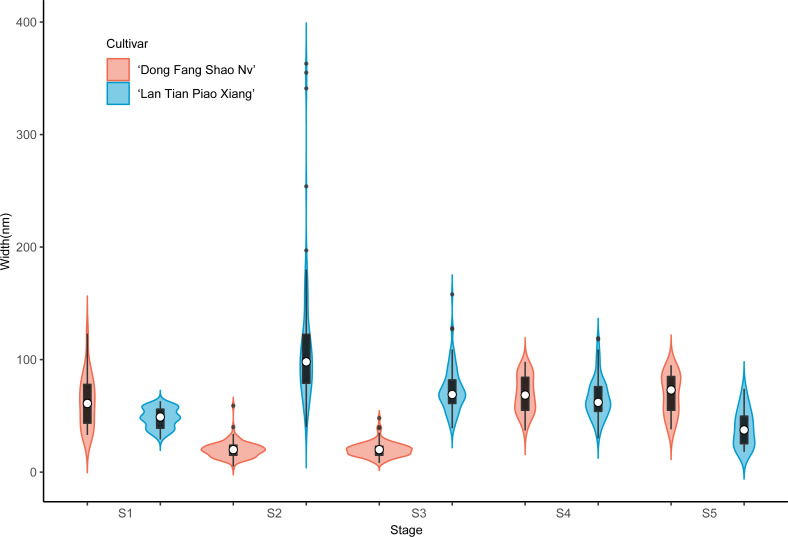
Contour width distributions of CSP samples isolated from herbaceous peony stem cell walls at S1–S5. The colored areas indicate the frequency distribution of the width. The vertical black bars indicate the range of the mean value, where the white solid dots represent the median of the width. Black solid bar above and below the white solid circle indicates the upper and lower quartile, respectively. The black scatter indicates outlier values in the data.

The violin plot of the width of the contoured backbone of two cultivars was used to characterize the individual molecules of CSP samples (Fig. 5). At the first three stages of development, the CSP width distributions of the two cultivars were distinctly different. In comparison to ‘Dong Fang Shao Nv’, at S1, the violin plot of ‘Lan Tian Piao Xiang’ was more compact, suggesting a more homogeneous CSP in width, which was reflected as the agglomeration structure in Fig. 3B. Furthermore, the CSP of ‘Lan Tian Piao Xiang’ was wider than that of ‘Dong Fang Shao Nv’ at S2–S3. The CSP width of ‘Lan Tian Piao Xiang’ was smaller than that of ‘Dong Fang Shao Nv’ at both S4 and S5 when stem bending was evident, as reflected in the reduction in the median in the violin plot.

**Figure 6:**
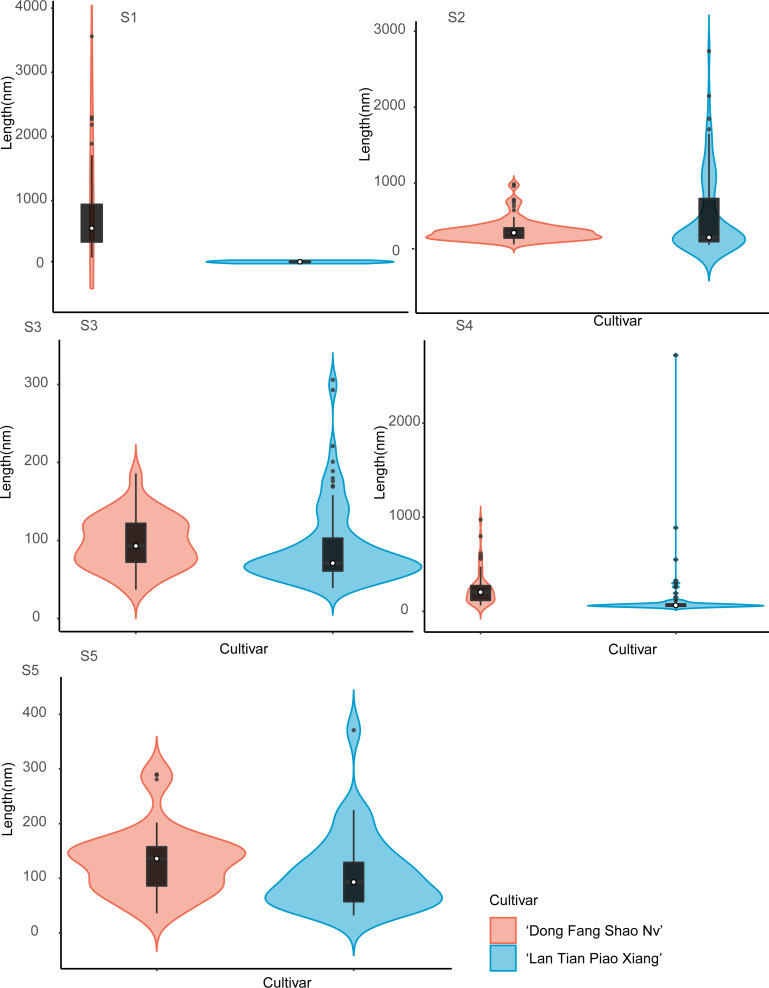
Distributions of chain length of CSP isolated from herbaceous peony stem cell walls at S1–S5. The colored areas indicate the frequency distribution of the length. The vertical black bars indicate the range of the mean value, where the white solid dots represent the median of the width. Black solid bar above and below the white solid circle indicates the upper and lower quartile, respectively. The black scatter indicates outlier values in the data.

The violin plot of the frequency distribution of the CSP length measurement is shown in Fig. 6. Because the pectin structure of ‘Dong Fang Shao Nv’ developed long and branched chains while ‘Lan Tian Piao Xiang’ still showed agglomeration, the shapes of the two cultivars of violin diagrams at S1 showed great differences. At S2–S4, although most of the CSP lengths of the two cultivars were similar, as reflected by the similar median values, the number of long chains was significantly higher in ‘Lan Tian Piao Xiang’ because of the larger outliers. There were more short chains and a wider variation of chain lengths in ‘Lan Tian Piao Xiang’ according to the violin graphs at S5.

**Figure 7:**
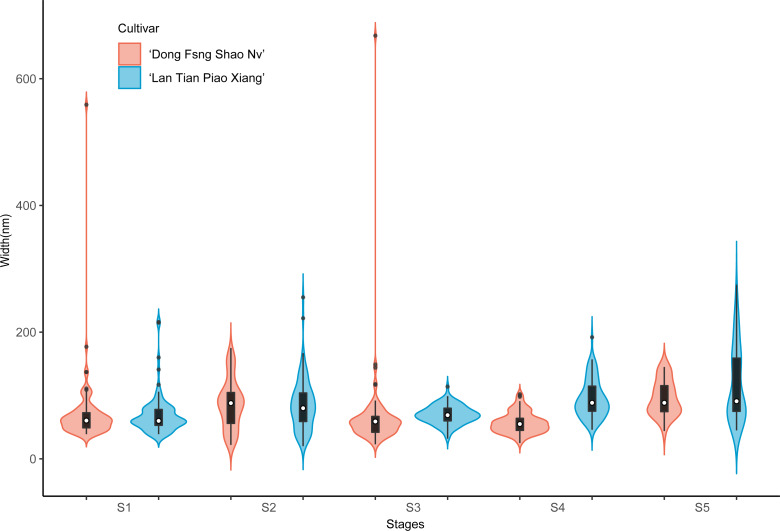
Contour width distributions of SSP samples isolated from herbaceous peony stem cell walls at S1–S5. The colored areas indicate the frequency distribution of the width. The vertical black bars indicate the range of the mean value, where the white solid dots represent the median of the width. Black solid bar above and below the white solid circle indicates the upper and lower quartile, respectively. The black scatter indicates outlier values in the data.

Contour widths of isolated polymer chains of SSP are measured at S1–S5 in ‘Dong Fang Shao Nv’ and ‘Lan Tian Piao Xiang’ and presented as violin plots in Fig. 7. The chain width distribution of both cultivars did not show much difference at S1–S3, except for the outliers with large values in ‘Dong Fang Shao Nv’ at S1 and S3. The violin plot distribution of the widths of the two cultivars at S4 and S5 indicated that ‘Lan Tian Piao Xiang’ had wider CSP chains.

**Figure 8:**
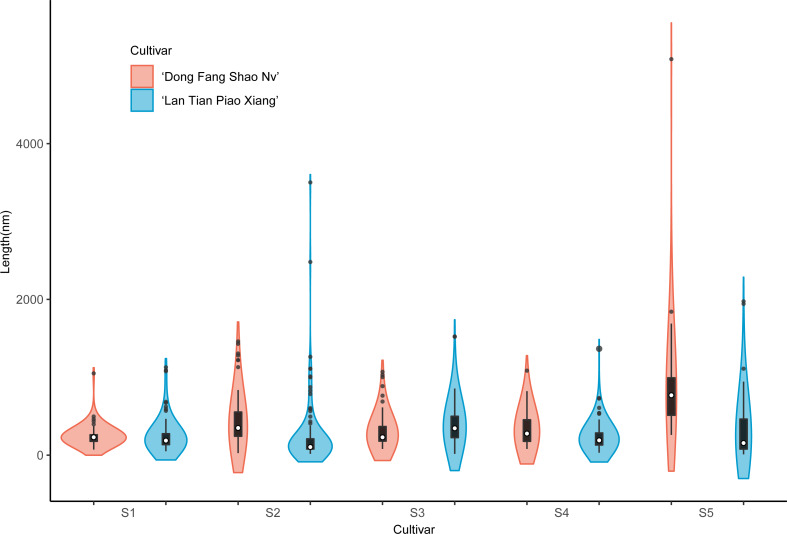
Distributions of chain and length of SSP isolated from herbaceous peony stem cell walls at S1–S5. The colored areas indicate the frequency distribution of the length. The vertical black bars indicate the range of the mean value, where the white solid dots represent the median of the width. Black solid bar above and below the white solid circle indicates the upper and lower quartile, respectively. The black scatter indicates outlier values in the data.

The violin plot in Fig. 8 shows the distribution of SSP lengths for the two cultivars. The shape of the violin plot was very similar for both cultivars at all stages. Except for S3, the median SSP chain length of ‘Lan Tian Piao Xiang’ was smaller than that of ‘Dong Fang Shao Nv’ at all other stages. Moreover, at S2–S4, ‘Lan Tian Piao Xiang’ had greater SSP long-chain outlier values.

## Discussion

Our previous studies have demonstrated that differences in straightness among herbaceous peony cultivars appear relatively early at S3 (bud conception stage, before buds show color). This manuscript focused on the WSP, CSP, and SSP contents, where the CSP and SSP contents of the two cultivars differed at each stage from S1 to S3. In contrast, both at the bud softness stage (S4) and the flowering stage (S5), there were significant differences in WSP and CSP contents between the two cultivars. Previous studies have mainly suggested that thickening of the secondary cell wall and lignin content are the main factors affecting the straightness of herbaceous peony stems (Zhao et al., 2020a; Tang et al., 2022). Our study implies that pectin, which mainly exists in the primary cell wall, is very likely to play an important role in supporting the mechanical strength of stems in herbaceous plants. This hypothesis can be further tested by subsequent studies.

Many studies in fruits have demonstrated that the nanostructure of pectin correlates with the mechanical strength of the cell wall (Cardenas-Perez et al., 2018; Deng et al., 2019; Wang et al., 2021). In this study, the nanostructure of pectin in the cell wall of herbaceous peony stems was studied for the first time by AFM, and the three-dimensional morphological images of three pectin monomers were generated at the single-molecule level with nanoscale resolution. WSP differs in width, and CSP and SSP differ in the length and width of nanostructures.

WSP gradually agglomerated, and two morphological structures, agglomerates, and linear chains, appeared during the development stage of herbaceous peony stems. Both structures were also observed in apricots (Deng et al., 2018). An increase in WSP agglomerates was observed during mango softening, presumably due to SSP transformation (Cardenas-Perez et al., 2018).

CSP exhibited agglomerate and chain-like morphology and was branched in the upright cultivar with a larger proportion of broad chains at later stages of development. Therefore, the more branched and wider CSP structure is more likely to be one of the influencing factors leading to the straightness of herbaceous peony. Similarly, the two forms of structure in CSP, which was referred to as “structural heterogeneity”, were also found in strawberry and apricot ripening, where the transformation of both structures might be related to hydrolase action (Zhang et al., 2018; Deng et al., 2019). Degradation of the CSP chain structure and reduction in width occurred with the storage softening process of strawberry fruit (Zhang et al., 2018). CSP is mainly located in the middle lamella and is rich in abundant homogalacturonan (HG), whose role is to increase intercellular adhesion by branched chains linked to adjacent cells in the form of ionic bonds (Wang et al., 2021). HG readily binds to calcium ions and it has been shown in strawberry that calcium treatment can inhibit the degradation of CSP, thereby maintaining the hardness of the fruit (Zhang et al., 2018; Huang et al., 2022). Previous studies on herbaceous peony found that EGTA treatment triggered the loss of Ca^2+^ in the cell wall and reduced stem strength, but the main focus was on the effect of lignin. Combined with the findings in this study, pectin may also play a role, which can be further explored in depth.

SSP consists of short chains, with branching side chains in the upright cultivar and wider and shorter chains in the bent cultivar. Therefore, the more stacked side chains and the narrower and longer chain SSP structure may help herbaceous peony to stay upright. Similarly, branching SSP chains were also observed in strawberries and apples (Paniagua et al., 2017b; Pieczywek et al., 2020), and small amounts of long chains were present in unripe strawberry fruits but not in ripe fruits (Paniagua et al., 2017b). During storage of plums at 0 °C, fruit hardness decreased, and SSP branching structures were reduced (Pan et al., 2018). SSP consists mainly of neutral, sugar-rich rhamnogalacturonan (RG) (Wang et al., 2021). As HG pectin is tougher than RG pectin, SSP does not form long, straight molecular chains like CSP (Pieczywek et al., 2020). The neutral sugar side chains in SSP may entangle with other cell wall polymers or bind to substances such as cellulose, thereby anchoring the RG to the wall (Maxwell et al., 2012; Paniagua et al., 2016). The loss of side chains affects the coagulation ability of pectin and weakens the cell wall cross-linkage network (Paniagua et al., 2017b; Pieczywek et al., 2020). RG-I is thought to be wrapped around the surface of microfibrils, thus enabling the interconnection of pectin and cellulose-polysaccharide cell wall networks (Vincken et al., 2003). Experiments with pectinase treatment of pear pectin fractions demonstrated that the degradation of pectin nanostructures was associated with the softening of cell walls, probably due to the formation of new pores and the increase in the pore size of the pectin matrix (Koziol et al., 2017).

## Conclusion

In conclusion, the WSP, CSP, and SSP contents differed significantly among the cultivars with contrasting straightness and were correlated with the angle of the flower and branch. In the upright cultivar, the WSP content was low at S2–S5, while CSP was high at S1–S5 and SSP was high at S1–S4. The morphology, chain length and width of the pectin with three solubilities on the nanoscale varied during development and stem bending. WSP showed both agglomerate and chain structures, and larger agglomerates led to stem bending. CSP had more branched structures, and the proportion of broad chains increased with development stage in the upright cultivar, while in ‘Lan Tian Piao Xiang’, the chains were formed by agglomerates. For SSP fractions, the upright cultivar had more branched side chains and stacked at later development stages, while the bending stem cultivar had a greater proportion of short and wide chains. Pectin is most likely also an important cell wall substance that affects the straightness of herbaceous peony stems.

##  Supplemental Information

10.7717/peerj.15166/supp-1 Supplemental Information 1 Description of field trials.

10.7717/peerj.15166/supp-2 Supplemental Information 2 The contents and length and width of WSP, CSP and SSP.

## Additional Information and Declarations

### Data Deposition

The following information was supplied regarding data availability: The raw measurements are available in the Supplementary Files.

## References

[ref-1] Adams EL, Kroon PA, Williamson G, Morris VJ (2003). Characterisation of heterogeneous arabinoxylans by direct imaging of individual molecules by atomic force microscopy. Carbohydrate Research.

[ref-2] Alikhani TT, Tabatabaei SJ, Torkashvand AM, Talei D (2021). Silica nanoparticles and calcium on the histological characteristics and stem bending in gerbera cut flower. Ornamental Horticulture.

[ref-3] Brummell DA (2006). Cell wall disassembly in ripening fruit. Functional Plant Biology.

[ref-4] Cardenas-Perez S, Chanona-Perez JJ, Guemes-Vera N, Cybulska J, Szymanska-Chargot M, Chylinska M, Koziol A, Gawkowska D, Pieczywek PM, Zdunek A (2018). Structural, mechanical and enzymatic study of pectin and cellulose during mango ripening. Carbohydrate Polymers.

[ref-5] Chen H, Fang R, Deng R, Li J (2021). The OsmiRNA166b-OsHox32 pair regulates mechanical strength of rice plants by modulating cell wall biosynthesis. Plant Biotechnology Journal.

[ref-6] Cheng G, Wang L, He S, Liu J, Huang H (2020). Involvement of pectin and hemicellulose depolymerization in cut gerbera flower stem bending during vase life. Postharvest Biology and Technology.

[ref-7] Daher FB, Braybrook SA (2015). How to let go: pectin and plant cell adhesion. Frontiers in Plant Science.

[ref-8] Deng LZ, Mujumdar AS, Yang XH, Wang J, Zhang Q, Zheng ZA, Gao ZJ, Xiao HW (2018). High humidity hot air impingement blanching (HHAIB) enhances drying rate and softens texture of apricot via cell wall pectin polysaccharides degradation and ultrastructure modification. Food Chemistry.

[ref-9] Deng LZ, Pan Z, Zhang Q, Liu ZL, Zhang Y, Meng JS, Gao ZJ, Xiao HW (2019). Effects of ripening stage on physicochemical properties, drying kinetics, pectin polysaccharides contents and nanostructure of apricots. Carbohydrate Polymers.

[ref-10] Fishman ML, Levaj B, Gillespie D, Scorza R (1993). Changes in the physico-chemical properties of peach fruit pectin during pn-tree ripening and storage. Journal of the American Society for Horticultural Science.

[ref-11] Frempong KEB, Chen Y, Wang Z, Xu J, Xu X, Cui W, Gong H, Peng D, Liang L, Meng Y, Lin X (2022). Study on textural changes and pectin degradation of tarocco blood Orange during storage. International Journal of Food Properties.

[ref-12] Hou JH, Wan YL, Liu AQ, Hong AY, Liu Y (2022). Content changes of cell wall composition during stem development in different varieties of *Paeonia lactiflora*. Acta Agriculturae Zhejiangensis.

[ref-13] Hu Y, Javed HH, Asghar MA, Peng X, Brestic M, Skalický M, Ghafoor AZ, Cheema HN, Zhang F-F, Wu Y-C (2022). Enhancement of lodging resistance and lignin content by application of organic carbon and silicon fertilization in *Brassica napus* L. Frontiers in Plant Science.

[ref-14] Huang W, Shi Y, Yan H, Wang H, Wu D, Grierson D, Chen K (2022). The calcium-mediated homogalacturonan pectin complexation in cell walls contributes the firmness increase in loquat fruit during postharvest storage. Journal of Advanced Research.

[ref-15] Kamenetsky-Goldstein R, Yu X (2022). Cut peony industry: the first 30 years of research and new horizons. Horticulture Research.

[ref-16] Koziol A, Cybulska J, Pieczywek PM, Zdunek A (2017). Changes of pectin nanostructure and cell wall stiffness induced in vitro by pectinase. Carbohydrate Polymers.

[ref-17] Li C (2013). Studies on physiological mechanism affecting the mechanical strength of inflorescence stem in herbaceous peony (*Paeonia lactiflora* Pall.). PhD dissertation.

[ref-18] Li X (2019). Evaluation on lodging resistance and mechanism of lodging resistance in sugarcane. PhD dissertation.

[ref-19] Liu L, Liu S, Lu H, Tian Z, Zhao H, Wei D, Wang S, Huang Z (2022). Integration of transcriptome and metabolome analyses reveals key lodging-resistance-related genes and metabolic pathways in maize. Frontiers in Genetics.

[ref-20] Maxwell EG, Belshaw NJ, Waldron KW, Morris VJ (2012). Pectin—an emerging new bioactive food polysaccharide. Trends in Food Science & Technology.

[ref-21] Miller CN, Harper AL, Trick M, Werner P, Waldron K, Bancroft I (2016). Elucidation of the genetic basis of variation for stem strength characteristics in bread wheat by Associative Transcriptomics. BMC Genomics.

[ref-22] Pan H, Wang L, Wang R, Xie F, Cao J (2018). Modifications of cell wall pectin in chilling-injured ‘Friar’ plum fruit subjected to intermediate storage temperatures. Food Chemistry.

[ref-23] Paniagua C, Blanco-Portales R, Barcelo-Munoz M, Garcia-Gago JA, Waldron KW, Quesada MA, Munoz-Blanco J, Mercado JA (2016). Antisense down-regulation of the strawberry beta-galactosidase gene *FabetaGal4* increases cell wall galactose levels and reduces fruit softening. Journal of Experimental Botany.

[ref-24] Paniagua C, Kirby AR, Gunning AP, Morris VJ, Matas AJ, Quesada MA, Mercado JA (2017a). Unravelling the nanostructure of strawberry fruit pectins by endo-polygalacturonase digestion and atomic force microscopy. Food Chemistry.

[ref-25] Paniagua C, Santiago-Domenech N, Kirby AR, Gunning AP, Morris VJ, Quesada MA, Matas AJ, Mercado JA (2017b). Structural changes in cell wall pectins during strawberry fruit development. Plant Physiology Biochemistry.

[ref-26] Pieczywek PM, Kozioł A, Płaziński W, Cybulska J, Zdunek A (2020). Resolving the nanostructure of sodium carbonate extracted pectins (DASP) from apple cell walls with atomic force microscopy and molecular dynamics. Food Hydrocolloids.

[ref-27] R Core Team (2022). http://www.r-project.org/.

[ref-28] Ren Y, Huang D, Liu S, Zhao F, Yu K, Zhu S (2022). Sodium hydrosulfide delays the softening of fig fruit during cold storage. Scientia Horticulturae.

[ref-29] Renard Catherine MGC (2005). Variability in cell wall preparations: quantification and comparison of common methods. Carbohydrate Polymers.

[ref-30] Satya P, Sarkar D, Vijayan J, Ray S, Ray DP, Mandal NA, Roy S, Sharma L, Bera A, Kar CS, Mitra J, Singh NK (2020). Pectin biosynthesis pathways are adapted to higher rhamnogalacturonan formation in lignocellulosic jute (*Corchorus* spp.). Plant Growth Regulation.

[ref-31] Shi Y, Li BJ, Su G, Zhang M, Grierson D, Chen KS (2022a). Transcriptional regulation of fleshy fruit texture. Journal of Integrative Plant Biology.

[ref-32] Shi Y, Man J, Huang Y, Zhang J, Zhang Z, Yin G, Wang X, Liu S, Chen Y, Wang X, Wei S (2022b). Overexpression of PnMYB2 from *Panax notoginseng* induces cellulose and lignin biosynthesis during cell wall formation. Planta.

[ref-33] Somerville CR, Bauer S, Brininstool G, Facette M, Hamann T, Milne J, Osborne E, Paradez A, Persson S, Raab TK (2004). Toward a systems approach to understanding plant cell walls. Science.

[ref-34] Tang Y, Shi W, Xia X, Zhao D, Wu Y, Tao J (2022). Morphological, microstructural and lignin-related responses of herbaceous peony stem to shading. Scientia Horticulturae.

[ref-35] Tang Y, Zhao D, Meng J, Tao J (2019). EGTA reduces the inflorescence stem mechanical strength of herbaceous peony by modifying secondary wall biosynthesis. Horticulture Research.

[ref-36] Uluisik S, Seymour GB (2020). Pectate lyases: their role in plants and importance in fruit ripening. Food Chemistry.

[ref-37] Vincken JP, Schols HA, Oomen RJ, McCann MC, Ulvskov P, Voragen AG, Visser RG (2003). If homogalacturonan were a side chain of rhamnogalacturonan I. Implications for cell wall architecture. Plant Physiology.

[ref-38] Wan Y, Zhang M, Hong A, Lan X, Yang H, Liu Y (2020). Transcriptome and weighted correlation network analyses provide insights into inflorescence stem straightness in *Paeonia lactiflora*. Plant Molecular Biology.

[ref-39] Wang H, Wang J, Mujumdar AS, Jin X, Liu ZL, Zhang Y, Xiao HW (2021). Effects of postharvest ripening on physicochemical properties, microstructure, cell wall polysaccharides contents (pectin, hemicellulose, cellulose) and nanostructure of kiwifruit (*Actinidia deliciosa*). Food Hydrocolloids.

[ref-40] Wang X, Shi X, Zhang R, Zhang K, Shao L, Xu T, Li D, Zhang D, Zhang J, Xia Y (2022). Impact of summer heat stress inducing physiological and biochemical responses in herbaceous peony cultivars (*Paeonia lactiflora* Pall.) from different latitudes. Industrial Crops and Products.

[ref-41] Xiao C, Barnes WJ, Zamil MS, Yi H, Puri VM, Anderson CT (2017). Activation tagging of Arabidopsis POLYGALACTURONASE INVOLVED IN EXPANSION2 promotes hypocotyl elongation, leaf expansion, stem lignification, mechanical stiffening, and lodging. The Plant Journal.

[ref-42] Zhang L, Zhao S, Lai S, Chen F, Yang H (2018). Combined effects of ultrasound and calcium on the chelate-soluble pectin and quality of strawberries during storage. Carbohydrate Polymers.

[ref-43] Zhang WW, Zhao SQ, Zhang LC, Xing Y, Jia WS (2020). Changes in the cell wall during fruit development and ripening in *Fragaria vesca*. Plant Physiology and Biochemistry.

[ref-44] Zhao D, Han C, Tao J, Wang J, Hao Z, Geng Q, Du B (2012). Effects of inflorescence stem structure and cell wall components on the mechanical strength of inflorescence stem in herbaceous peony. International Journal of Molecular Sciences.

[ref-45] Zhao D, Hao Z, Tao J, Han C (2013). Silicon application enhances the mechanical strength of inflorescence stem in herbaceous peony (*Paeonia lactiflora* Pall.). Scientia Horticulturae.

[ref-46] Zhao D, Luan Y, Shi W, Tang Y, Huang X, Tao J (2022). Melatonin enhances stem strength by increasing the lignin content and secondary cell wall thickness in herbaceous peony. Journal of Experimental Botany.

[ref-47] Zhao D, Luan Y, Xia X, Shi W, Tang Y, Tao J (2020a). Lignin provides mechanical support to herbaceous peony (*Paeonia lactiflora* Pall.) stems. Horticulture Research.

[ref-48] Zhao D, Shi W, Xia X, Tang Y, Tao J (2020b). Microstructural and lignin characteristics in herbaceous peony cultivars with different stem strengths. Postharvest Biology and Technology.

[ref-49] Zhao D, Tang Y, Xia X, Sun J, Meng J, Shang J, Tao J (2019). Integration of transcriptome, proteome, and metabolome provides insights into how calcium enhances the mechanical strength of herbaceous peony inflorescence stems. Cell.

[ref-50] Zhao D, Xu C, Luan Y, Shi W, Tang Y, Tao J (2021). Silicon enhances stem strength by promoting lignin accumulation in herbaceous peony (*Paeonia lactiflora* Pall.). International Journal of Biological Macromolecules.

[ref-51] Zhao L, Liu A, Zhang J, Han J, Liu Y (2015). Study on the stem orthostatic performance of *Paeonia lactiflora* under facility cultivation. Acta Agriculturae Zhejiangensis.

[ref-52] Zhou H (2017). Study on the evaluation of ramie lodging resistance and loding resistance traits. Master’s Degree Dissertation.

