# Supplementary material for: Effect of the pectin contents and nanostructure on the stem straightness of two Paeonia lactiflora cultivars
Source: PeerJ. 2023 Apr 13;11:e15166. doi: 10.7717/peerj.15166 (PMC10106084; doi:10.7717/peerj.15166)
Supplement: Supplemental Information 1 [file peerj-11-15166-s001.docx]

Description of field trials

The plants involved in this manuscript were obtained from plants growing naturally in the base of our unit and were sampled only, without spraying drugs or undergoing genetic modification. Therefore, no field permission is required.
